# Efficacy of IFN-λ1 to Protect Human Airway Epithelial Cells against Human Rhinovirus 1B Infection

**DOI:** 10.1371/journal.pone.0095134

**Published:** 2014-04-21

**Authors:** Fahad Gulraiz, Carla Bellinghausen, Mieke A. Dentener, Niki L. Reynaert, Giel R. Gaajetaan, Erik V. Beuken, Gernot G. Rohde, Cathrien A. Bruggeman, Frank R. Stassen

**Affiliations:** 1 Department of Medical Microbiology, Maastricht University Medical Centre, Maastricht, the Netherlands; 2 Department of Respiratory Medicine, Maastricht University Medical Centre, Maastricht, the Netherlands; Kantonal Hospital St. Gallen, Switzerland

## Abstract

Impaired interferon (IFN) production has been observed in various obstructive respiratory diseases. This contributes to enhanced sensitivity towards viral infections triggering acute exacerbations. To compensate for this impaired host IFN response, there is need to explore new therapeutic strategies, like exogenous administration of IFNs as prophylactic treatment. In the present study, we examined the protective potential of IFN-λ1 and compared it with the previously established protecting effect of IFN-β. A549 cells and human primary bronchial epithelial cells were first treated with either IFN-β (500 IU/ml) or IFN-λ1 (500 ng/ml) for 18 h. For infection, two approaches were adopted: i) Continuous scenario: after pre-treatment, cells were infected immediately for 24 h with human rhinovirus 1B (HRV1B) in IFN-containing medium, or were cultured for another 72 h in IFN-containing medium, and then infected for 24 h with HRV1B, ii) Pre-treatment scenario: IFN-containing medium was replaced after 18 h and cells were infected for 4 h either immediately after pre-treatment or after additional culturing for 72 h in IFN-free medium. The protective effect was evaluated in terms of reduction in the number of viral copies/infectious progeny, and enhanced expression of IFN-stimulated genes (ISGs). In both cell types and in both approaches, IFN-λ1 and IFN-β treatment resulted in pronounced and long-lasting antiviral effects exemplified by significantly reduced viral copy numbers and diminished infectious progeny. This was associated with strong up-regulation of multiple ISGs. However, in contrast to the IFN-β induced expression of ISGs, which decreased over time, expression of ISGs induced by IFN-λ1 was sustained or even increased over time. Here we demonstrate that the protective potential of IFN-λ1 is comparable to IFN-β. Yet, the long-lasting induction of ISGs by IFN-λ1 and most likely less incitement of side effects due to more localized expression of its receptors could make it an even more promising candidate for prophylactic treatment than IFN-β.

## Introduction

Acute exacerbations are the major cause of morbidity and mortality in chronic respiratory diseases like asthma and chronic obstructive pulmonary disease (COPD). Also, they increase the economic burden because of extra healthcare, which has to be provided to the patients [1], [2]. Among others, viral infections, especially infections with human rhinovirus (HRV), are strongly implicated as important triggers for the induction of acute exacerbations [3]–[6]. Following HRV infections, healthy individuals develop upper respiratory symptoms (common cold) but patients with chronic respiratory diseases frequently develop more severe lower respiratory tract symptoms [7], [8].

The airway epithelium provides the first line of defense against invading pathogens. In response to viral infections, airway epithelial cells become activated and start producing different antiviral mediators and pro-inflammatory cytokines. These mediators and cytokines not only combat invading viruses, but also recruit and activate other immune cells and initiate mechanisms of adaptive immunity [9], [10]. Three different types of interferons (type-I [IFN-α/β], type-II [IFN-γ] and the more recently discovered type-III [IFN-λ]) are among the most important antiviral mediators produced by epithelial cells. Although all three types have antiviral properties, type-I and type-III are the IFNs which are produced in direct response to viral infection [11]–[14]. Nonetheless, type-III IFNs are considered to be more important for mucosal antiviral defense, while type-I IFNs might be more important for clearance of systemic viral infections [15], [16].

Viral infections result in the activation of transcription factors like nuclear factor kappaB (NFκB) and interferon regulatory factor (IRF) –3 and IRF-7, which regulate the production of IFNs at the transcriptional level. Yet, different subtypes of IFNs respond differently to IRF-3 and IRF-7. Transcription of IFN-λ1 (type-III) and IFN-β (type-I) genes is controlled by both IRF-3 and IRF-7, while transcription of other subtypes genes (type-III IFN-λ2/3 and type-I IFN-α) is predominantly regulated by IRF-7 [17]. This differential regulation plays an important role in the kinetics of induction of different subtypes. IRF-3 is constitutively and ubiquitously expressed in human cells. Due to this, when activated upon viral entry, it up-regulates the expression of IFN-λ1 and IFN-β. In contrast, IRF-7 is not constitutively expressed in most cells and is induced in response to IFNs. Because of this IFN-λ1 and IFN-β behave as early response genes while IFN-λ2/3 and IFN-α genes are expressed with delayed kinetics [18], [19].

Deficient production of IFNs has been observed in cells isolated from asthma and COPD patients upon HRV infections [8], [20]. This indicates an impaired antiviral response which makes these patients more susceptible to viral infections and which may ultimately lead to the induction of acute exacerbations. Unfortunately, currently available strategies and therapies for the prevention and treatment of virus-induced acute exacerbations have limited efficacy [21] and new options need to be explored. One potential therapeutic strategy could be the prophylactic exogenous administration of IFNs.

In a previous *in vitro* study, we have shown that exogenous application of low amounts of IFN-β induces pronounced and long-lasting protective effects against HRV infections in human respiratory epithelial cells [22]. Alternatively, despite the increasing recognition of the importance of type-III IFN in airway antiviral defense [9], [16], [23], [24], their potential as prophylactic agents still needs to be evaluated. In the present study we evaluated the potential protective effects of IFN-λ1 against HRV1B infection in airway epithelial cells and compared it with the protective effects of IFN-β.

## Materials and Methods

### Cell Culture

A549 cells (ATCC CCL-185; Rockville, MD, USA) were cultured in RPMI 1640 medium (Invitrogen, Grand Island, NY, USA) supplemented with 10% fetal calf serum (FCS; Lonza, Verviers, Belgium) and incubated at 37°C/5% CO_2_.

Primary bronchial epithelial cells (PBECs) were isolated from bronchus rings obtained from patients who underwent surgery for solitary pulmonary nodules. Lung tissues used for isolation were located at the remotest possible distance from the nodule and were macroscopically cancer free. PBECs were isolated and cultured as previously described [25]. Tissue was obtained from the Maastricht Pathology Tissue Collection (MPTC). Collection, storage and use of tissue and patient data were performed in agreement with the “Code for Proper Secondary Use of Human Tissue in the Netherlands” (http://www.fmwv.nl). Patient’s characteristics are summarized in table S1.

MRC5 cells (ATCC CCL-171) were maintained in EMEM (Invitrogen, Grand Island, NY, USA) supplemented with non-essential amino acids (MP Biomedicals, Solon, Ohio, USA) L-glutamine (2 mM) and 10% FCS (Lonza, Verviers, Belgium). Cells were incubated at 37°C/5% CO_2_.

### Ethics Statement

After reviewing the protocol within the context of the Medical Research Involving Human Subjects Act, the local Medical Ethics Committee of the Maastricht University Medical Center (METC azM/UM) waived the need for ethical approval or informed consent.

### Virus Culture

HRV1B was purchased from American Type Culture Collection (ATCC, VR-1645). Viral stocks were generated as previously described [22]. Briefly, MRC5 were infected in EMEM with 2% FCS, non-essential amino acids, L-glutamine (2 mM). Once 100% cytopathogenic effect (CPE) was obtained, cell debris was removed by centrifugation and viral titers were determined by TCID50 (50% tissue culture infectivity dose) on MRC-5 cells.

Respiratory syncytial virus A2 (RSV) was obtained from the Netherlands Vaccine institute and propagated on Vero cells. After 2 h of infection, cells were washed and incubated further in DMEM with 1% FCS until >80% CPE was attained. Cell debris was removed by centrifugation for 10 minutes at 1000×g. The virus pool was then precipitated using polyethylene glycol (PEG): PEG stock (50% PEG6000 in 150 mM NaCl, 1 mM EDTA, 6.1 g/L TRIS, pH 7.5), was mixed with the virus pool to achieve a final concentration of 10% PEG (1∶5), stirred 2 h at 4°C, centrifuged 30 min at 3000×g and the pellet was resuspended in PBS +25% sucrose (10% of original volume = 10×concentrated). Viral titers were determined by TCID50.

### Determination of Cytotoxicity of IFNs

Because in some experiments cells were cultured for more than 100 hours in the continuous presence of either IFN-β or IFN-λ1, cytotoxic effects of both cytokines were determined with a colorimetric thiazolyl blue tetrazolium bromide (MTT) (Sigma-Aldrich, St. Louis, MO, USA) assay as described previously [22]. Briefly, A549 cells were seeded in a 96-well plate and exposed for 114 h to IFN-β (500 IU/ml) or IFN-λ1 (500 ng/ml). Afterwards the MTT assay was performed according to the manufacturer’s instructions. The percentage of metabolic activity of exposed A549 cells was calculated by comparing to non-exposed controls (absorbance exposed/absorbance non-exposed×100%).

### Protective Effects of IFN-β/−λ1 Treatment

Recombinant human IFN-β and IFN-λ1 were purchased from PBL Biomedical Laboratories (NJ, USA). In order to determine the maximum protective potential of the two IFNs, dose-dependent induction of ISGs was determined in A549 cells. Maximal expression of ISGs was achieved at 500 IU/ml for IFN-β and at 500 ng/ml for IFN-λ1 (Fig. S1). These doses of the respective IFNs were therefore used in all further experiments. The protective effect of IFNs against HRV infections was examined in 2 different experimental approaches (Fig. S2). In all cases, A549 cells were seeded in 24-well tissue culture plates (Becton Dickinson, NJ, USA) at a density of 2×10^5^ cells per well in RPMI 1640 (10% FCS). After 24 h, the medium was replaced by fresh RPMI 1640 (2% FCS) and the experiments were started.

#### IFN pre-treatment

In the first approach, cells were pre-treated with either IFN-λ1 or IFN-β for 18 h. Then IFN-containing medium was replaced with fresh IFN-free medium and cells were infected immediately with HRV1B at a multiplicity of infection of 1 (MOI-1) for 4 h at 33°C/5%CO_2_. Also, to examine how long the protective effect of the pre-treatment was maintained, we incubated cells for another 72 h after pre-treatment before infection. After 4 h of infection, HRV1B containing medium was removed and replaced by fresh IFN-free medium. Cells were cultured for another 24 h and then collected and processed for further analyses.

#### Continuous exposure

Although the approach described above may reveal important information regarding the antiviral properties of the two types of IFN, it may not reflect the natural course of both treatment and infection. Therefore we also performed a series of experiments in which neither the cytokine nor the virus was removed once added. Thus, after the 18 h pre-treatment period, HRV1B (MOI-1) was added to the cells in the continuous presence of either IFN-λ1 or IFN-β and cells were cultured for another 24 h in the presence of IFN-β, IFNβ+HRV1B, IFNλ1 or IFNλ1+HRV1B. After this infection period cells were washed and collected for further analyses. Also, the long-lasting effect of IFN-β or IFN-λ1 treatment was examined when cells were infected after 72 h of IFN treatment.

For PBECs, cells were seeded in Greiner 24-well plates in BD medium (contains 50% DMEM (Gibco) and 50% bronchial epithelial basal medium (BEBM, Lonza) supplemented with BEGM SingleQuots (Lonza) and BSA (1.5 µg/ml; Sigma). When cell layers were approximately 80% confluent, growth medium was replaced with BD starvation medium (BD medium without EGF, BPE, BSA and gentamicin) and the treatment with IFNs was initiated 24 h later. Cells were then treated for 18 h with either IFN-λ1 or IFN-β and subsequently infected with HRV1B as described above. In PBECs the long-lasting effect of either pre-treatment or continuous treatment could not be examined because of deterioration of cell viability after 72 h of culturing.

### RSV Infection and Poly (I: C)/LyoVec Treatment

With the purpose of determining the induction of IFN expression in response to stimuli other than HRV1B, cells were infected with RSV at MOI-1 for one hour at 37°C or stimulated with 500 ng/ml of poly (I:C)/Lyovec (low molecular weight) (Invitrogen) and incubated at 37°C/5%CO_2_ for 24 h. With poly (I:C)/Lyovec, A549 were stimulated for 18 h. Afterwards cells were collected and processed for further analyses.

### TCID50

Virus titration assay was performed as previously described [26] with some modifications. Supernatants from infected PBECs were collected and serially diluted 1∶2 in MEM containing 2% FCS (Lonza, Verviers, Belgium), non-essential amino acids and L-glutamine (2 mM). Dilutions were added on confluent MRC-5 in 96-well plates. Each dilution was assayed in six wells and TCID50 was calculated according to Spearman-Karber formula.

### Plasmid Construction for the Generation of Standard Curves

For determination of the actual number of viral RNA copies present in the cells, a plasmid was constructed as previously described [27] with some modifications. In summary, after reverse transcription of viral RNA, a PCR was performed. The PCR fragment was cloned into the pGEMT-easy vector (Promega) and sequenced. The resulting plasmid, designated p1123, was transcribed *in* *vitro* and the resulting RNA was quantified. Serial dilutions of the quantified RNA were used for the generation of a standard curve.

### Quantification of virus Load in Infected Cells

Total RNA was isolated with RNeasy kit (Qiagen, Hilden, Germany) according to manufacturer’s instructions. After DNase treatment (Turbo DNA-free kit, Ambion, Austin, TX, USA), the amount of RNA in the samples was quantified with a Nanodrop ND-1000 and 1 µg of RNA was reverse transcribed into cDNA using the iScript cDNA Synthesis kit (Bio-Rad, Hercules, CA, USA). qPCR was performed to amplify cDNA as described previously [28]. For HRV and the IFN genes, cDNA was amplified in a volume of 25 µl containing IQ Supermix (Bio-Rad, Hercules, CA, USA). For all other genes, HOT FIREPol EvaGreen qPCR mix plus (Solis Biodyne, Tartu, Estonia) was used. The sequences of the primers are given in table S2. For determination of viral RNA copy numbers, standard curves were generated for every qPCR run along with the samples. Gene expression was normalized to β-actin level and fold changes were calculated by using 2^−ΔΔCt^ method. In case basal expression of a gene was not detectable, expression was expressed using the 2^−ΔCt^ method [29].

### Statistical Analysis

The overall significance of the experimental effect was determined by Kruskal-Wallis tests. Once overall significance was achieved, the differences between multiple groups were analyzed by Mann-Whitney test. Values of *p*<0.05 were considered statistically significant for both Kruskal-Wallis test and Mann-Whitney test. Data are expressed as mean ± SEM.

## Results

### Long Term Exposure to Recombinant IFNs has No Toxic Effects on A549 Cells

Since our experimental design involved long-term exposure of cells to IFNs, possible toxic effects of this exposure were determined first. For this, cells were exposed to IFNs for about 114 h, which was the maximum time for which the cells were to be exposed to IFNs in our main experiments. After that, metabolic activity of cells was determined by MTT test as an indication of cellular viability. No significant differences were observed between IFNs exposed and non-exposed cells. This indicates that long term IFNs exposure has no negative effect on the viability of cells (Fig. S3).

### IFN-λ1 Induces Strong Antiviral State in A549 Cells

**Figure 1:**
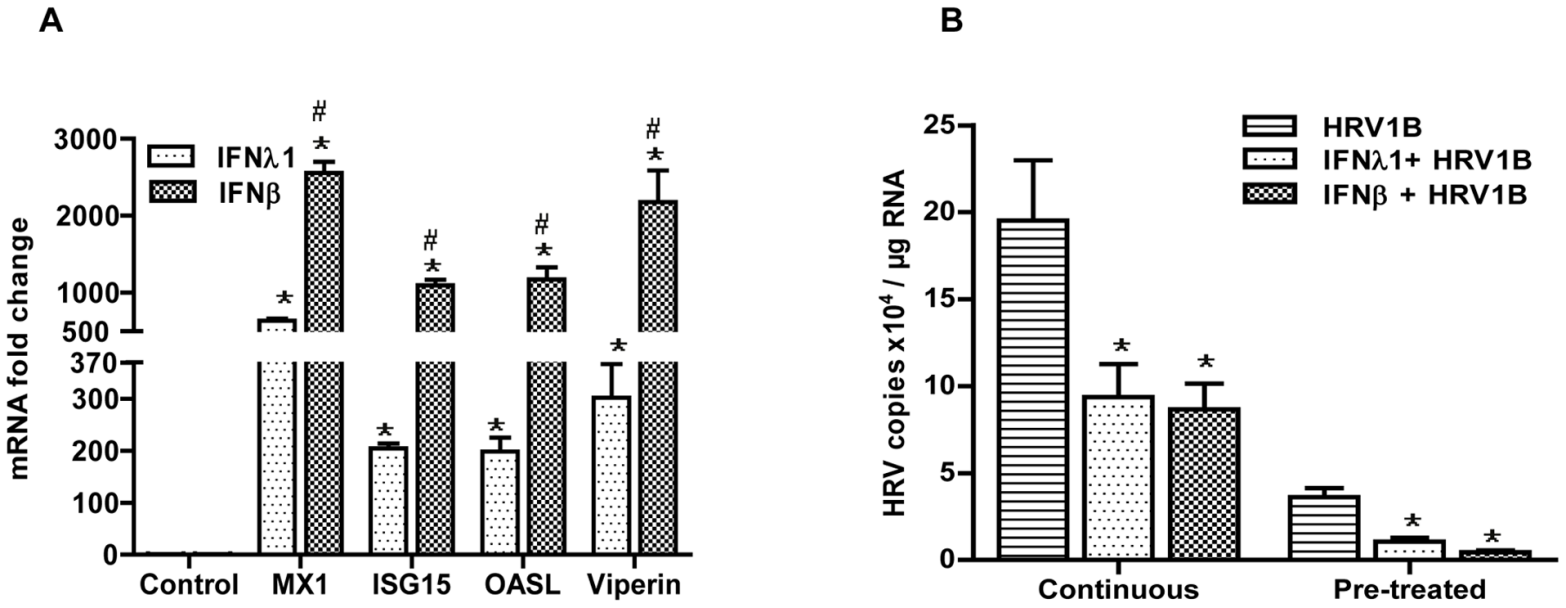
IFN-induced antiviral state protects against HRV1B infection in A549. A549 were treated with IFNs for 18(n = 6). Fold-changes were calculated with the 2^−ΔΔCt^ method (A). A549 were first treated with IFNs for 18 h. Next, according to the continuous and pre-treatment approaches, cells were infected with HRV1B and incubated for another 24 h (n = 6). After that, cells were collected for further analyses. Viral copies were determined by qPCR and fold-changes were calculated with the 2^−ΔΔCt^ method (B). *, p<0.05 Control vs IFNs, HRV1B vs IFNs+HRV1B. #p<0.05 IFN-λ1 vs IFN-β.

In our previous study we have shown that exogenous administration of IFN-β induced a strong up-regulation of ISGs in respiratory epithelial cells [22]. Previously, ISGs have been shown to contribute significantly to antiviral defense [30]. Here we demonstrate that *in vitro* treatment of A549 cells for 18 h with IFN-λ1 likewise induces a significant up-regulation of various ISGs (Fig. 1A). Though our initial dose-response evaluation experiments showed maximum induction of ISGs mRNA levels at 500 ng/ml of IFN-λ1, they were significantly lower as compared to IFN-β. Therefore, in the following experiments we examined whether the IFN-λ1 induced up-regulation of ISGs is sufficient to protect cells against a subsequent viral infection.

### IFN-λ1-induced Antiviral State Protects against HRV1B Infection

Next we tested the antiviral potency of IFN-λ1 and compared it to the previously demonstrated potency of IFN-β. Therefore A549 cells were first treated with IFNs for 18 h and then infected with HRV1B according to two different protocols.

#### Pre-treatment only

First, and in line with other papers demonstrating a protective effect of IFNs [31], we only pre-treated cells for 18 h with either IFN-λ1 or IFN-β, removed the IFN containing medium and then infected them for 4 h with HRV1B. After another 24 h, cells were collected and HRV1B RNA copy numbers were determined. Figure 1B clearly demonstrates that both IFNs protected the cells against HRV1B infection, as HRV RNA copy numbers were significantly lower in IFN pre-treated cells when compared to untreated cells. This decrease in viral copies tended to be more pronounced in IFN-β treated samples when compared to IFN-λ1, which corresponds with a more pronounced up-regulation of ISGs in IFN-β treated samples as compared to IFN- λ1 (Fig. 1B).

#### Continuous exposure

Although pre-treatment followed by an infection for a limited time is a well-accepted model in literature, it may not reflect the natural course of both treatment and infection. Therefore, in an attempt to mimic the *in vivo* situation more closely, we first treated cells with either of the IFNs for 18 h and subsequently infected them with HRV1B in the continuous presence of the IFNs. Also, virus was not removed after 4 h as in the pre-treatment protocol, but was kept present in the incubation medium until cells were collected (after 24 h). As expected, at 24 h after infection viral copy numbers were markedly higher in the untreated cells when compared to cells infected for only 4 h (4 h: 3.6×10^4^ vs. 24 h: 2.0×10^5^ copies/µg RNA). Nevertheless, despite these higher viral copy numbers, both types of IFNs were still able to reduce the number of HRV RNA copies significantly (Fig. 1B).

### IFN-λ1-induced Antiviral State Provides Long-lasting Protection against HRV1B Infection

Previously, we have shown that exogenous administration of IFN-β provides a long-lasting protection against viral infection [22]. After confirming that cells were protected against a viral infection immediately after IFN-λ1 treatment, we aimed to explore whether IFN-λ1 also has the potential to provide a long-lasting protection. A549 cells were therefore treated with IFN-λ1 or IFN-β for 18 h only. Then the IFN-containing medium was either washed away (pre-treatment only) and cells were cultured for another 72 h in the absence of IFNs, or cells were cultured for another 72 h in the continuous presence of either one of the IFNs (continuous exposure).

**Figure 2:**
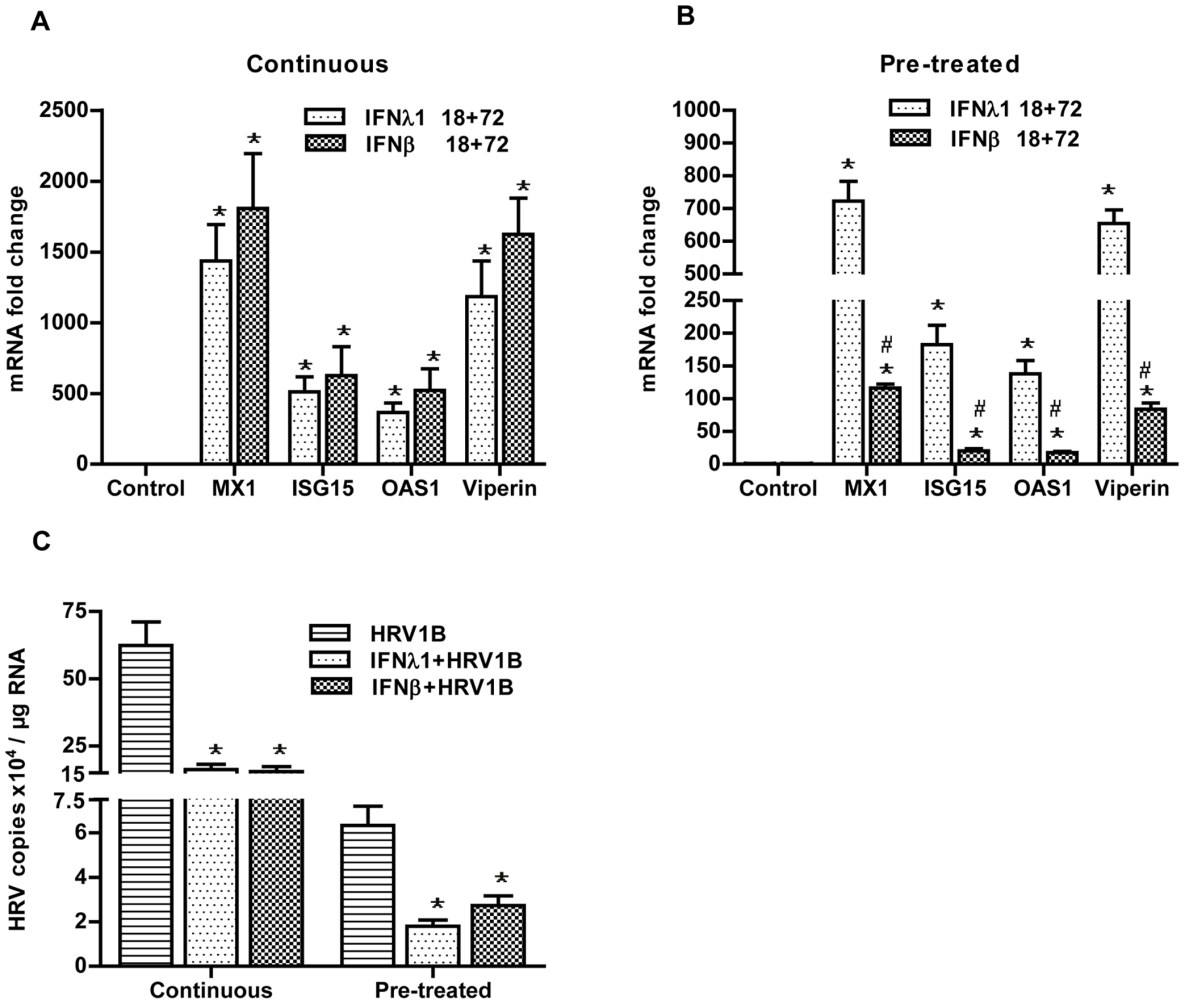
Long-lasting antiviral state induced by IFNs. A549 were treated with IFNs for 18-treatment approaches, cells cultured for another 72 h and collected afterwards for further analyses (n = 4). mRNA expression of different genes was determined by qPCR and fold changes were calculated with the 2^−ΔΔCt^ method (A & B). Moreover, to evaluate the antiviral status of the cells after 72 h of culturing, cells were infected with HRV1B and incubated for another 24 h (n = 5). After that, viral copies were determined by qPCR and fold changes were calculated with the 2^−ΔΔCt^ method (C). *, p<0.05 HRV1B vs IFNs+HRV1B, control vs IFNs. #, p<0.05 IFN-λ1 vs IFN-β.

To check whether cells were still in an antiviral state, we determined the expression of the various ISGs under different conditions. As expected, prolonged continuous exposure to IFNs resulted in high levels of all ISGs determined, and no differences were observed between IFN-λ1 and IFN-β (Fig. 2A). Also when cells were only pre-treated for 18 h, ISG levels were still significantly enhanced even after additional culturing for 72 h in the absence of the IFNs. Surprisingly, ISG levels were significantly higher in cells pre-treated with IFN-λ1, suggesting that the protective effects of this type III IFN may last longer than the effect of IFN-β (Fig. 2B).

To test whether this antiviral state, either after continuous exposure or pre-treatment only, was sufficient to protect cells against HRV infection, cells were infected with HRV1B for either 4 h (pre-treatment protocol, cells collected after another 24 h) or for 24 h (continuous protocol) 72 h after the initial IFN treatment. Figure 2C shows that in both conditions a significant decrease in viral copies was found in IFN-treated cells as compared to control cells. These data imply that a single treatment with IFN-λ1, like IFN-β, is able to induce an antiviral state in these cells, which protects them for a prolonged time (in this case 72 h) against future viral infections.

### Priming of Cells with IFNs Enhances their Ability to Produce IFNs

IFNs can act in an autocrine and/or paracrine way to up-regulate their own expression, thereby reinforcing and/or maintaining their antiviral effects (“priming”). In order to determine whether the observed long-lasting protective effect was due to this phenomenon, we determined whether treatment with either IFN-β or -λ1 resulted in such a (cross-) priming effect. Also, we assessed whether the initial IFN treatment followed by an HRV1B infection affected the induction of IFN-β/-λ1 expression. Cells were therefore initially treated with either IFN-β or -λ1 for 18 h and cultured for another 72 h according to the pre-treatment or continuous protocol. Afterwards cells were either infected with HRV1B or not and cultured for another 24 h.

### Expression of IFN-β after Stimulation with IFNs

**Figure 3:**
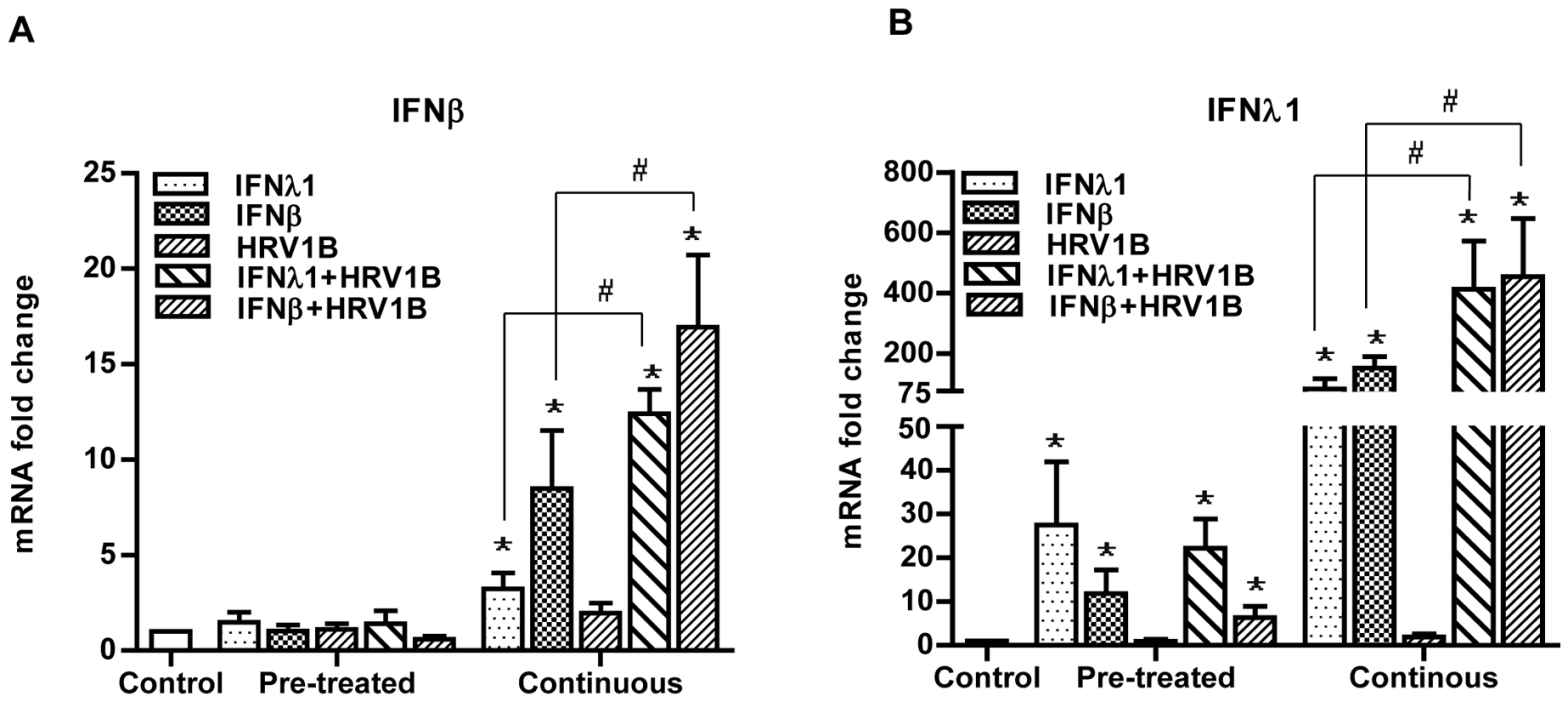
Priming effect of IFNs on HRV1B-induced interferon response. A549 were first treated with IFNs for 18-treatment approaches. After that cells were infected with HRV1B for 4 h in the same medium. After the infection period, either same medium was maintained on the cells (continuous) or replaced by fresh medium (pre-treated). Thereafter, cells were incubated for another 24 h and then collected for further analyses. mRNA levels of IFN-β (A) and IFN-λ1 (B) were determined by qPCR and fold changes were calculated with the 2^−ΔΔCt^ method. HRV1B induced average IFN-β expression was 1.8 (continuous) and 0.99 (pre-treated) folds and average IFN-λ1 expression was 1.92 (continuous) and 0.90 folds (pre-treated). *, p<0.05 control vs rest of conditions. #, p<0.05 IFN vs IFN+HRV1B. n = 4.

Although it has been shown previously that IFN-β priming enhanced the expression of IFN-β [32], we were unable to see this effect when cells were only pre-treated (Fig. 3A). Also, no clear evidence for cross-priming was observed as IFN-λ1 pre-treatment did not have any significant effect on the expression of IFN-β mRNA. Surprisingly, subsequent infection with HRV1B also did not enhance the expression of IFN-β, irrespective of whether cells were pre-treated with IFN-β or IFN-λ1. Alternatively, when cells were stimulated with IFNs for the entire period, the expression of IFN-β mRNA was significantly enhanced both after treatment with IFN-β (8.5-fold) or IFN-λ1 (3.2-fold) (p<0.005), indicating that (cross-)priming occurred after prolonged exposure. Moreover, when cells were continuously stimulated and subsequently infected with HRV1B, the expression of IFN-β mRNA was further enhanced irrespective of the type of IFN they were first treated with (16.1-fold with IFN-β and 12.4-fold with IFN-λ1 (p≤0.05) (Fig. 3A).

### Expression of IFN-λ1 after Stimulation with IFNs

We also examined whether priming resulted in enhanced expression of IFN-λ1 (Fig. 3B). In contrast to priming with IFN-β, we noticed a significant increase in the expression of IFN-λ1 mRNA even when cells were only pre-treated for 18 h and collected at the end of the experiment (27.5-fold with IFN-λ1 and 11.8-fold with IFN-β) (p<0.01). However, no further increase was observed when cells were additionally infected with HRV1B (22.0-fold with IFN-λ1 and 6.3-fold with IFN-β). On the other hand, continuous stimulation with either of both cytokines was sufficient for a significant induction of the expression of IFN-λ1 mRNA (90-fold with IFN-λ1 and 180-fold with IFN-β) (p<0.005), which was further increased as a result of HRV1B infection (460-fold with IFN-λ1 and 513-fold with IFN-β) (p<0.05).

Summarizing, we noticed a strong (cross-) priming effect of IFN-β and IFN-λ1 on the expression of IFN-λ1. In contrast, IFN-β seemed less prone to (cross-)priming.

### HRV1B does not Induce an Interferon Response

**Figure 4:**
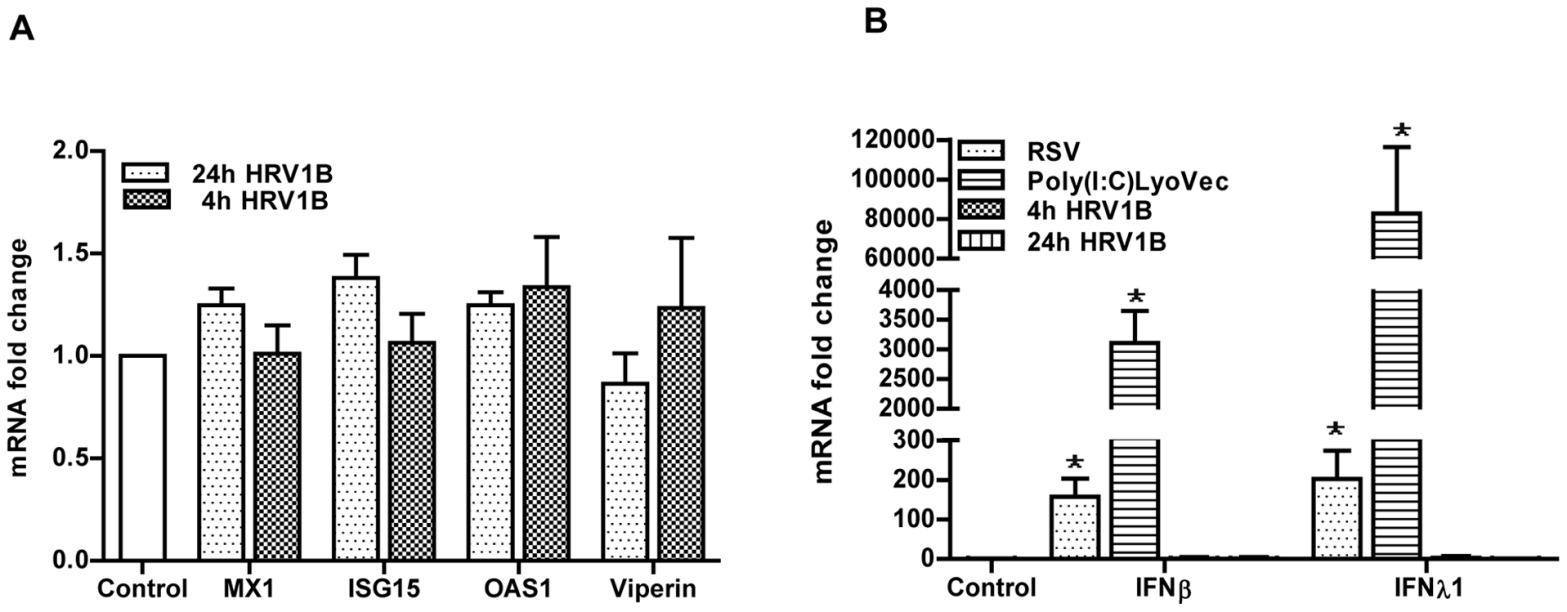
HRV1B induced interferon response in A549. A549 were infected with HRV1B for 4(n = 6). The mRNA expression of ISGs was determined with qPCR and fold changes were calculated with the 2^−ΔΔCt^ method (A). Cells were infected with RSV (MOI-1, 1 h) or HRV1B (4 h). After infection, virus containing medium was removed and replaced with fresh medium. Then cells were collected after 24 h (n = 5). While with poly(I:C)/LyoVec (500 ng/ml), cells were collected after 18 h of stimulation (n = 3). IFNβ/λ1 mRNA expression was determined by qPCR and fold changes were calculated with the 2^−ΔΔCt^ method (B). *, p<0.05 control vs rest of the conditions.

In the previous experiment we intriguingly noticed that the expression of IFN-β was hardly enhanced when cells were infected with HRV1B. Usually, basal IFNs and ISGs mRNA levels are very low in uninfected cells and it is generally assumed that their expression strongly increases after viral infection. Accordingly, previous reports demonstrated an increase in the expression of both type I and III IFN mRNA in epithelial cells following HRV infection [33], [34]. Surprisingly, however, we did not observe such an increase in the expression of these genes in the experiments described above. To further explore this observation, we infected A549 cells for 4 h and determined the expression of IFN-β/-λ1 and various ISGs after 24 h. However, as already shown in experiments described above, no induction of any of the ISGs was observed even when HRV1B was continuously present for the entire 24 h period (Fig. 4A). Similarly, no change in IFN-β expression was found while IFN-λ1 levels remained undetectable. Infection with another ssRNA virus, RSV, or stimulation with the viral mimic polyI:C/LyoVec both resulted in the induction of IFN genes, indicating these cells are well able to produce type I and III IFNs (Fig. 4B).

### IFN-λ1 Induces an Antiviral State in PBECs which Provides Protection against HRV1B Infection

**Figure 5:**
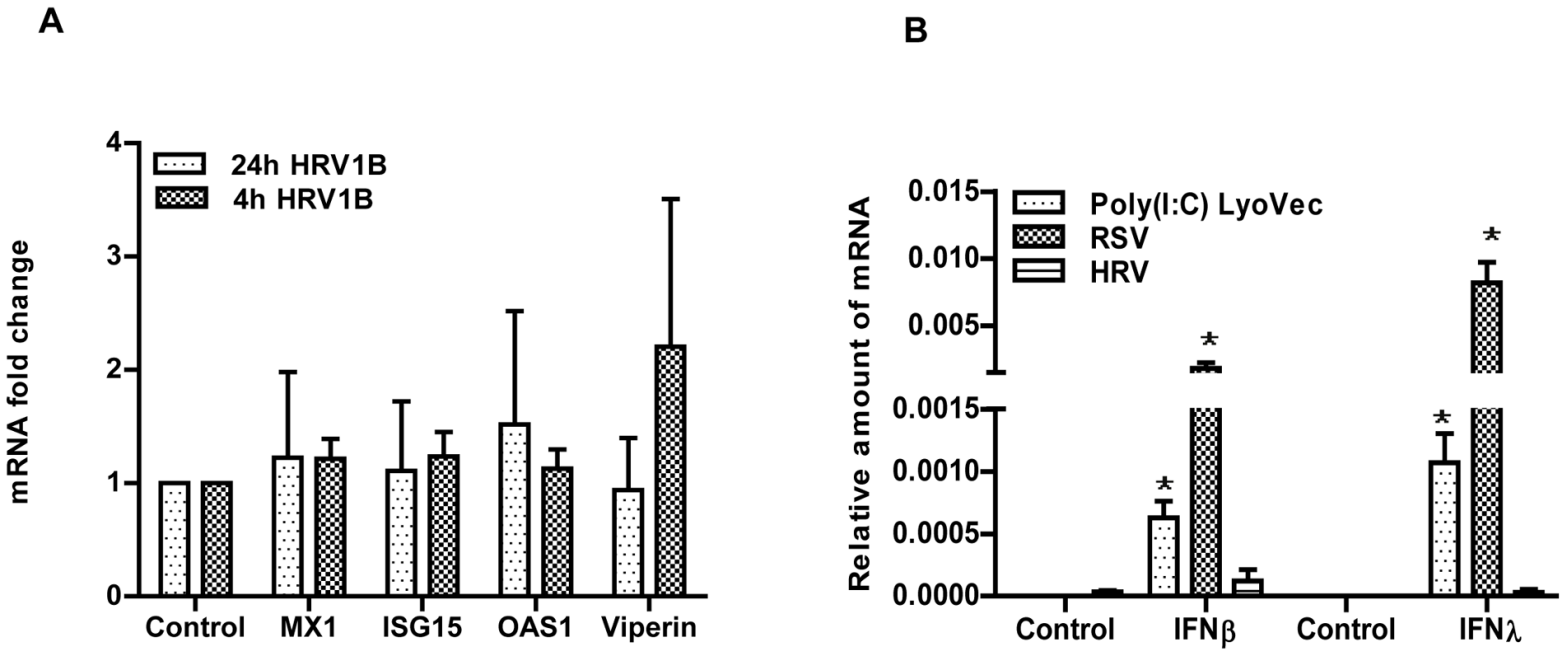
HRV1B induced interferon response in PBECs and its comparison with other stimuli. PBECs were infected with HRV1B for 4(n = 4). The mRNA expression of ISGs was determined with qPCR and fold changes were calculated with the 2^−ΔΔCt^ method (A). Cells were infected with RSV (MOI-1, 1 h) or HRV1B (4 h). After infection, virus-containing medium was removed and replaced with fresh medium. Additionally, cells were stimulated with poly(I:C)/LyoVec (500 ng/ml) for 24 h (n = 3). Then cells were collected and IFNβ/λ1 mRNA expression was determined by qPCR and relative amount of mRNA was calculated with the 2^−ΔCt^ method (B). *, p<0.05 control vs rest of the conditions.

Although A549 cells are basically human alveolar epithelial cells, they were originally derived from an alveolar adenocarcinoma, and it can therefore not be excluded that they respond differently to external stimuli than primary cells. Moreover, A549 cells are of alveolar origin while bronchial epithelial cells come in contact with infectious agents before alveolar cells. Therefore, to put our results with A549 cells into perspective, we repeated some of the experiments with PBECs. First we also infected these cells with HRV1B for either 4 or 24 h and in line with our results found in A549 cells, we were unable to detect an up-regulation of IFN-β, IFN-λ1 or any of the ISGs. Nonetheless, similar to A549, PBECs were also able to express IFNs in response to stimulation with poly (I: C)/LyoVec or to a viral infection as RSV (Fig. 5A, B). The results both in A549 as well as the primary cells strongly suggest that HRV1B is to some extent able to evade the host antiviral response in our experimental settings.

**Figure 6:**
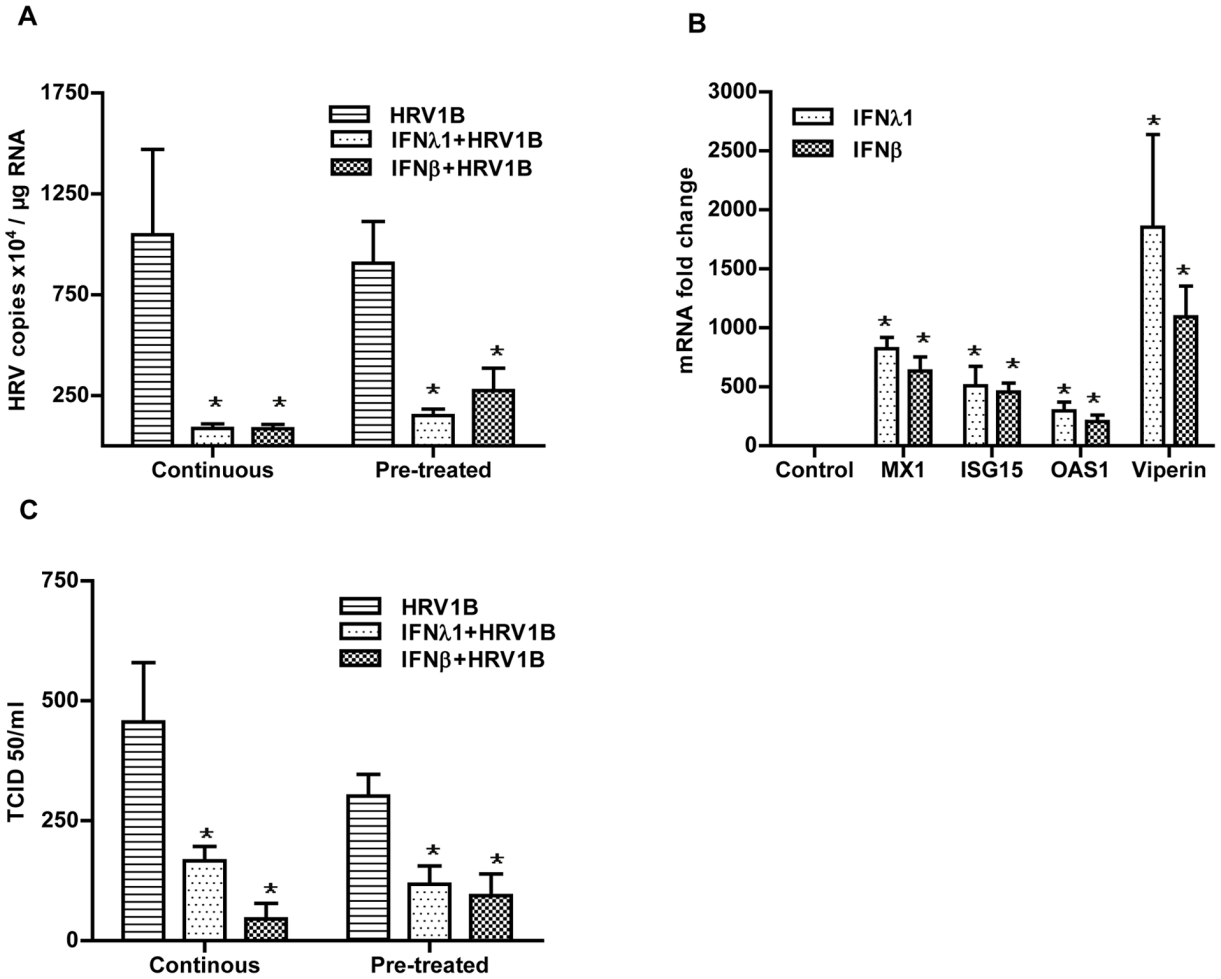
IFNs-induced antiviral state protects against HRV1B infection in PBECs. PBECs were infected with HRV1B for 4(continuous) or replaced with fresh medium (pre-treated). After that cells were incubated for another 24 h. Then the supernatant was collected to determine TCID50 (C) while cells were collected to determine viral copies (A). PBECs were treated with IFNs for 18 h and then mRNA expression of different ISGs was determined by qPCR. Fold changes were calculated with the 2^−ΔΔCt^ method (B). *, p<0.05 control vs rest of the conditions n = 4.

Next, we tested whether IFN treatment was able to protect primary cells against HRV infection. Therefore, cells were pre-treated for 18 h and then infected with HRV1B according to the pre-treatment or continuous protocol. Figure 6A shows that, like in A549 cells, both IFNs were well able to protect PBECs against HRV1B infection as viral RNA levels were noticeably reduced in pre-treated cells when compared to control cells. In line with this is the pronounced up-regulation of ISGs as shown in figure 6B. To further confirm the protective effect of IFN pre-treatment, we determined the generation of infectious viral particles. Again, and in accordance with viral RNA copy numbers, generation of infectious viral particles in IFN-treated cells was significantly lower as compared to non-treated samples (Fig. 6C). It should be mentioned that we were unable to test whether IFNs also had a long-lasting protective effect in PBEC because of viability issues when primary cells were cultured for more than 72 h regardless of IFN treatment.

## Discussion

In this study we assessed the efficacy of IFN-λ1 to protect respiratory epithelial cells against HRV1B infection and compared its effects to the previously reported protective effects induced by IFN-β. Results of the current study revealed that exogenous administration of both IFNs, irrespective of how cells had been treated (pre-treatment vs. continuous), induced a strong antiviral state in both A549 and PBECs, which was associated with a robust up-regulation of various ISGs. This antiviral state provided efficient protection against subsequent viral infection in both conditions. Yet, as compared to IFN-β, the IFN-λ1- induced antiviral state seemed to strengthen itself over time, as the expression of ISGs induced by IFN-λ1 was either sustained or even increased, while expression of IFN-β-induced ISGs decreased over time.

Type-III IFNs are relatively recently discovered members of the IFN family. Like type-I IFNs, type-III IFNs have also been shown to be induced by viral infections and to possess antiviral properties. Although type-I and type-III IFNs act through different receptors, both types of IFNs activate similar intracellular signaling pathways and because of this, they largely have similar biological activities. Both of them up-regulate the expression of ISGs, which are presumably responsible to provide antiviral resistance to the cells [35], [36]. However, not all types of cells respond to type-III IFNs, which is due to the fact that expression of receptors for type-III IFNs is limited to a few types of cells. Epithelial cells in the respiratory, gastrointestinal and reproductive tract are considered to be the primary cells which express receptors for type-III IFNs [15], [37]. Particularly in the respiratory tract, type-III IFNs have been shown to be important mediators in response to respiratory viral infections [16], [23], [24]. In this study we investigated whether type III IFNs have a potential as prophylactic agents against viral infections.

We and others have previously demonstrated that IFN-β treatment of respiratory epithelial cells induces a strong antiviral state in these cells which markedly protects them against viral infections. This antiviral condition was noticeably associated with a distinct up-regulation in the expression of various ISGs. In the present study we demonstrated similar effects when cells were treated with IFN-λ1 resulting in a 200- to 700-fold up-regulation in the expression of all ISGs examined. Although this up-regulation was significantly less than the effects induced by IFN-β, it was still sufficient to protect the cells from a subsequent HRV1B infection. Interestingly, in primary cells the effects of both IFNs were quite comparable, as no significant differences were found in the up-regulation of all ISGs, the presence of viral copies in the cells or the release of infectious progeny.

Induction of ISGs by type-I and –III IFNs is mainly dependent on the activation of the Jak/STAT signal transduction pathway [36], [38]. Differences in the activation of the Jak/STAT pathway by type-I and type-III IFNs have already been reported. Earlier, Maher and colleagues [39] have shown that continuous treatment of human keratinocyte cell line HaCat with IFN-λ1 resulted in sustained activation of STAT1 and STAT2 over the course of 24 h. On the contrary, treatment with IFN-α, a type-I IFN, resulted in transient activation of STAT1 and STAT2. This could be due to differential regulation of type-I and type-III signaling. They also showed that expression of IFN-λ1 induced ISGs continued to increase at 24 h. In the present study, we also observed differences in the kinetics of induction of ISGs by IFN-β and IFN-λ1. The protective effect induced by an 18 h pre-treatment with both types of IFNs was persistent and long-lasting. Even when cells were cultured in the absence of the IFNs for an additional 72 h after the 18 h pretreatment period, cells were significantly protected against a subsequent HRV infection. However, when cells were pre-treated with IFN-β, the expression of most of the ISGs peaked immediately after the 18 h pre-treatment period and declined significantly when cells were cultured for another 72 h in the absence of IFN-β. In contrast, when cells were pre-treated with IFN-λ1, the expression of all ISGs remained constant or was even increased after an additional 72 h of culturing in the absence of IFN-λ1. We did not determine the activation status of STAT1/STAT2, yet data of mRNA expression of IFNs presented in figure 3 show that the observed expression of ISGs is in line with the expression of IFNs. Also, it shows that IFN-λ1, in contrast to IFN-β, is well able to induce its own expression. This supports the findings of Ank and colleagues who demonstrated that both type I and type III IFNs are able to (cross-)induce the expression of type III IFNs but not type I IFNs [12]. However, in contrast to Ank et. al., we also observed an up-regulation of the IFN-β expression in response to long term stimulation with both IFN-β itself and also with IFN-λ1, both in the presence or absence of infectious agent HRV1B (continuous scenario). In the presence of HRV1B the increase in the IFN-β expression could be mediated by IRF-7, as both IFN-β and -λ1 can prime IRF-7, which will subsequently result in virus-induced nuclear translocation and increased transcription of IFNs [18], [32], [40]. Nonetheless, in the absence of a virus, the increase in the IFN-β expression could be driven by IRF-1. IRF-1 can be up-regulated by IFNs and then can induce IFN-β [41], [42]. Due to this characteristic of IFN-λ1, the strength of the antiviral state induced by this type of IFN may be maintained for a prolonged period.

In order to replicate in host cells, viruses have evolved different strategies to subvert the host interferon response. Also HRVs have been shown to have this ability. For example, when A549 cells were infected with HRV14 only low levels of IFN-β mRNA were induced as compared to those induced by vesicular stomatitis virus [43]. This attenuation of the antiviral response by the virus was shown to be due to interference with IRF3 activation. Similar results were found when HeLa cells were infected with HRV1a [44]. Consistent with these observations, one very recent study also showed no induction of IFN-β after HRV1B infection in primary bronchial epithelial cells, which were isolated from healthy persons [45]. Likewise, in the present study, we also did not observe an up-regulation of the IFNs or ISGs mRNA expression after infection of A549 or PBECs with HRV1B. In contrast, when PBECS were infected with RSV or were stimulated with poly(I:C)/LyoVec, a synthetic ligand for intracellular sensors of viral RNA, RIG-I and MDA5, we found a strong up-regulation in the expression of both IFN-β and IFN-λ1 mRNA. Similar results were observed in A549 cells. This indicates that cells are well able to respond to viral stimuli and are capable to mount both a type-I and a type-III IFN response. Furthermore, up-regulation of ISGs in response to IFN treatment shows that IFN signaling in these cells was also intact. These data suggest that HRV is able to actively interfere with the IFN-dependent antiviral response in respiratory epithelial cells. Nevertheless, this finding is in conflict with data published by several other groups who demonstrated a moderate to strong up-regulation of IFN-β mRNA expression [34], [46]. The reason for this discrepancy is not clear, but could be due to different experimental conditions, different virus strains/stocks or different types or sources of cells. In this study we did not further explore this inconsistency between different studies as it was beyond the scope of the present study, but further investigations are required to unravel the reason for these differences.

Altogether, these data indicate that both IFN-β and IFN-λ1 are able to provide protection against viral infection with comparable efficacy. However, because of the more restricted distribution of its receptors to the epithelial lining in particular of the respiratory tract, and a sustained long-term induction of ISGs, IFN-λ1 therapy can have a more important role in the prevention of viral infections with probable causation of fewer side effects. Yet, further work is required in particular regarding the efficacy of the cytokine in an *in vivo* setting.

## Supporting Information

Figure S1 **Dose titration of IFNs.** A549 were treated with IFN-β (25, 100, 500 and 1000 IU/ml) (A) and IFN-λ1 (25, 100 and 500 ng/ml) (B) for 18 h. mRNA expression of different ISGs was determined by qPCR (n = 4). Fold-changes were calculated with the 2^−ΔΔCt^ method.
(TIF)

Figure S2 **Schematic representation of treatment with IFNs and HRV1B infection protocol.** Cells were first treated with IFNs for 18 h. After that, two different approaches were followed for infection with HRV1B: i) Pre-treated: IFNs containing medium was replaced with fresh medium. Next, for immediate subsequent infection, cells were infected with HRV1B for 4 h (A), while for determining the long-lasting protective effect, cells were incubated for another 72 h and then infected with HRV1B for 4 h (C). After infection period, virus-containing medium was replaced with fresh medium and cells were incubated for another 24 h. ii) Continuous: After 18 h treatment with IFNs, for immediate subsequent infection cells were infected with HRV1B in the same medium for another 24 h (B) while to determine long-lasting protective effect cells were infected after 72 h in the same medium (D) and incubated for another 24 h.
(TIF)

Figure S3 **Toxicity of IFNs in A549 cells.** A549 cells were exposed to IFN-λ1 (500 ng/ml) or IFN-β (500 IU/ml) for 114 h. Metabolic activity of IFN-treated and non-treated cells was determined by MTT assay and compared to each other. Data are represented as mean +/− SEM of three independent experiments.
(TIF)

Table S1 (DOCX)

Table S2 (DOCX)
