# Supplementary figures and images for: Efficacy of IFN-λ1 to Protect Human Airway Epithelial Cells against Human Rhinovirus 1B Infection
Source: PLoS One. 2014 Apr 21;9(4):e95134. doi: 10.1371/journal.pone.0095134 (PMC3994020; doi:10.1371/journal.pone.0095134)

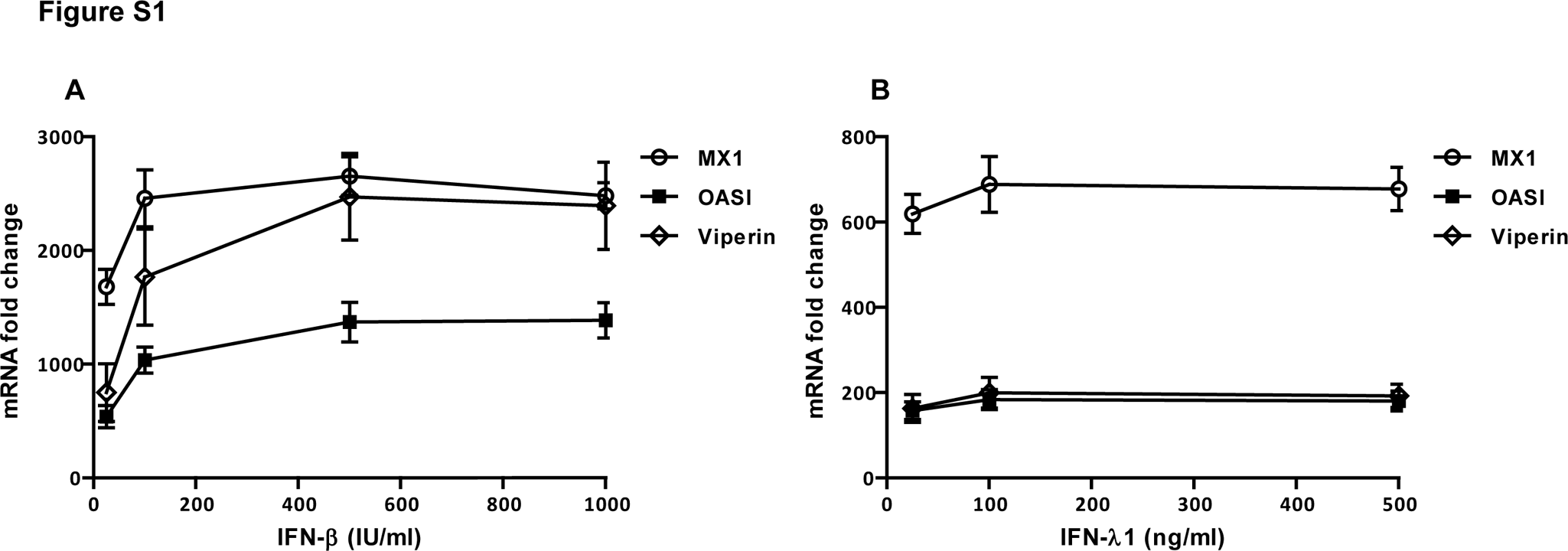

Supplement: Figure S1 — Dose titration of IFNs. A549 were treated with IFN-β (25, 100, 500 and 1000 IU/ml) (A) and IFN-λ1 (25, 100 and 500 ng/ml) (B) for 18 h. mRNA expression of different ISGs was determined by qPCR (n = 4). Fold-changes were calculated with the 2−ΔΔCt method. (TIF) [file pone.0095134.s001.tif]

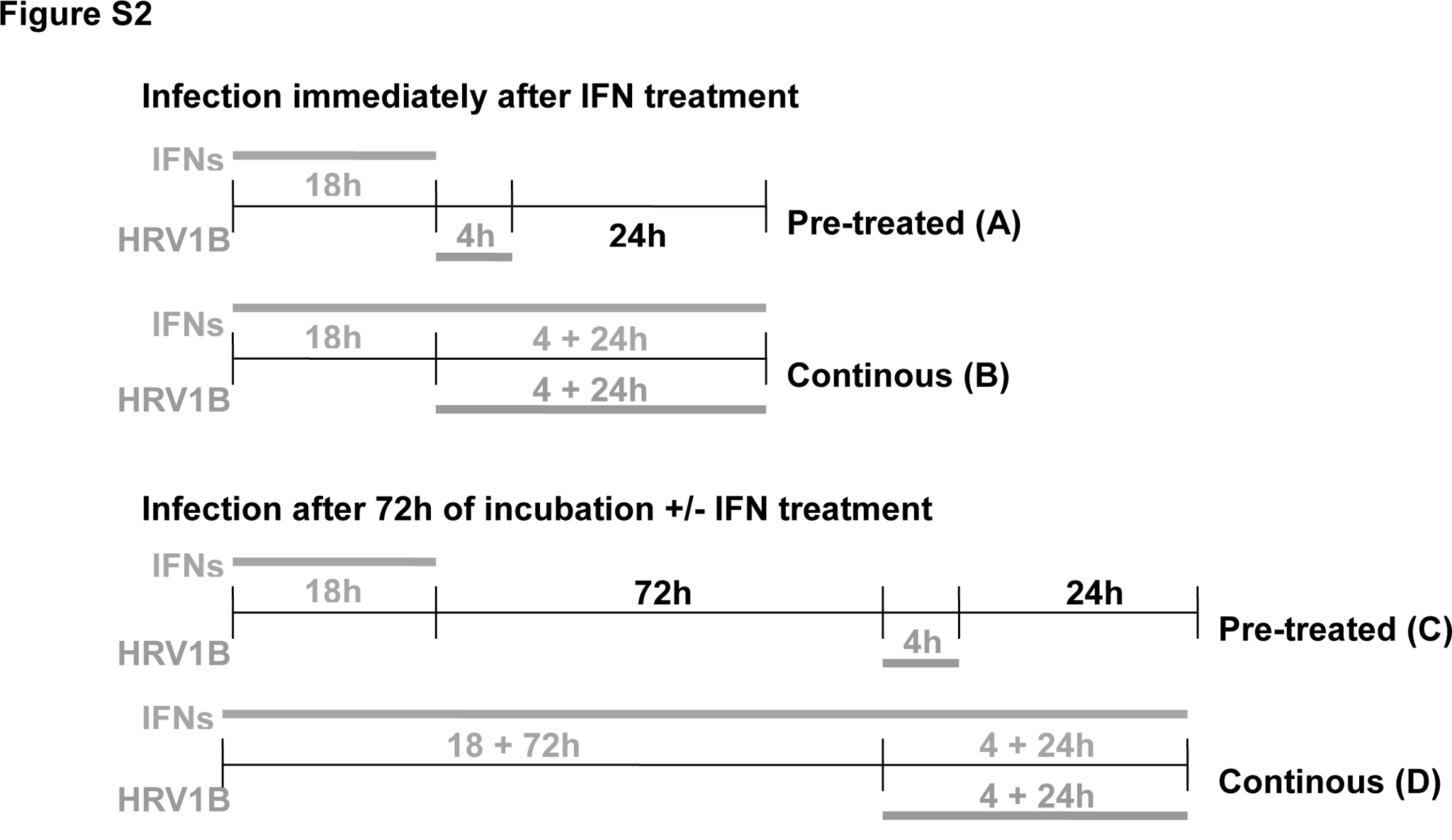

Supplement: Figure S2 — Schematic representation of treatment with IFNs and HRV1B infection protocol. Cells were first treated with IFNs for 18 h. After that, two different approaches were followed for infection with HRV1B: i) Pre-treated: IFNs containing medium was replaced with fresh medium. Next, for immediate subsequent infection, cells were infected with HRV1B for 4 h (A), while for determining the long-lasting protective effect, cells were incubated for another 72 h and then infected with HRV1B for 4 h (C). After infection period, virus-containing medium was replaced with fresh medium and cells were incubated for another 24 h. ii) Continuous: After 18 h treatment with IFNs, for immediate subsequent infection cells were infected with HRV1B in the same medium for another 24 h (B) while to determine long-lasting protective effect cells were infected after 72 h in the same medium (D) and incubated for another 24 h. (TIF) [file pone.0095134.s002.tif]

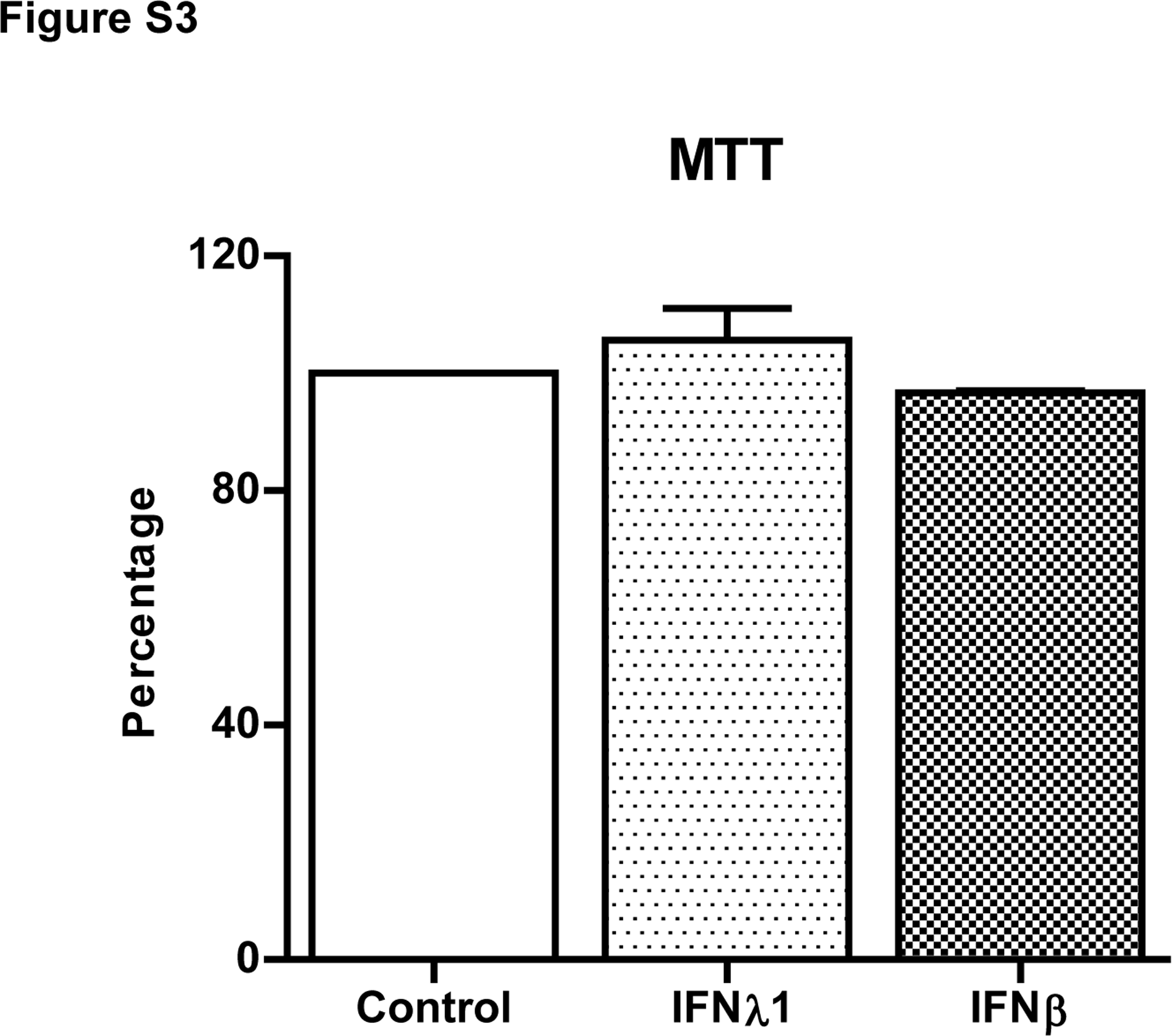

Supplement: Figure S3 — Toxicity of IFNs in A549 cells. A549 cells were exposed to IFN-λ1 (500 ng/ml) or IFN-β (500 IU/ml) for 114 h. Metabolic activity of IFN-treated and non-treated cells was determined by MTT assay and compared to each other. Data are represented as mean +/− SEM of three independent experiments. (TIF) [file pone.0095134.s003.tif]
