# Supplementary material for: Efficacy of IFN-λ1 to Protect Human Airway Epithelial Cells against Human Rhinovirus 1B Infection
Source: PLoS One. 2014 Apr 21;9(4):e95134. doi: 10.1371/journal.pone.0095134 (PMC3994020; doi:10.1371/journal.pone.0095134)
Supplement: Table S1 — (DOCX) [file pone.0095134.s004.docx]

**Table S1. Patient characteristics**

| Number of patients | 4 |
| --- | --- |
| Age in Years | 69.5 (65 – 75) |
| BMI (kg/m^2^) | 27.8 (20 – 35.26) |
| Sex (F = female, M = male) | F:1 : M:3 |
| Current smokers | 0 |
| Second-hand smoker exposure | 1 |
| Ex-smoker | 1 |
| Pack-years smoked | 15 (0 – 40) |
| FEV1 in % predicted | 114.6 (87.8 – 137.2) |
| FVCin in % predicted | 114.1 (77.7 – 141.3) |
| Tiffeneau-Index | 82.9 (74.8 – 105) |

BMI = Body Mass Index; FEV1 = Forced expiratory volume in 1 second; FVCin = inspiratory forced vital capacity
