# Supplementary material for: Efficacy of IFN-λ1 to Protect Human Airway Epithelial Cells against Human Rhinovirus 1B Infection
Source: PLoS One. 2014 Apr 21;9(4):e95134. doi: 10.1371/journal.pone.0095134 (PMC3994020; doi:10.1371/journal.pone.0095134)
Supplement: Table S2 — (DOCX) [file pone.0095134.s005.docx]

**Table S2. Primers used for mRNA expression analysis**

Gene Primer sequences (5’-3’)

HRV forward TGGACAGGGTGTGAAGAGC

reverse CAAAGTAGTCGGTCCCATCC

ISG15 forward GGTGGTGGACAAGTGCGATG

reverse CGAAGGTCAGCCAGAACAGG

Mx1 forward GGACATCGCCACCACAGAGG

reverse TCCGCACCACATCCACAACC

OASL forward TGGGATCTTCTCCCACACTC

reverse ATAGATCCCCAGACCCAACC

Viperin forward AAGCGCATATATTTCATCCAGAATAAG

reverse CACAAAGAAGTGTCCTGCTTGGT

IFN-β forward AAATTGCTCTCCTGTTGTGC

reverse TGCAGCTGCTTAATCTCCTC

IFN-λ1 forward GGACGCCTTGGAAGAGTCACT

reverse AGAAGCCTCAGGTCCCAATTC

β-actin forward TGGAGAAATCTGGCACCAC

reverse GAGGCGTACAGGGATAGCAC
